# Accuracy of flight time and countermovement-jump height estimated from videos at different frame rates with *MyJump*

**DOI:** 10.5114/biolsport.2023.118023

**Published:** 2022-09-06

**Authors:** Basilio Pueo, Will G Hopkins, Alfonso Penichet-Tomas, Jose M Jimenez-Olmedo

**Affiliations:** 1 Physical Education and Sports, Faculty of Education, University of Alicante, 03690 Alicante, Spain; 2 College of Sport and Exercise Science, Victoria University, Melbourne, Australia

**Keywords:** Athletes, Linear mixed model, Measurement error, Reliability, Smartphone app

## Abstract

Recent improvements in smartphone video technology may provide sufficient accuracy for estimation of jump height via flight time determined from video recordings of vertical-jump tests. The aim of this study is to evaluate the accuracy of jump height estimated from videos at different frame rates. High-definition videos of 10 young adults (6 males, 4 females) performing 5 countermovement jumps were recorded at a frame rate of 1000 Hz and transcoded to frame rates of 120, 240, and 480 Hz. Flight time in the videos was assessed independently by three observers at each of the four frame rates with MyJump. Flight time and jump height were analyzed with mixed models for estimation of means and of standard deviations representing technical error of measurement (free of within-subject jump-to-jump variability) at each frame rate. The four frame rates and three observers produced practically identical estimates of mean jump height. The technical errors at 120, 240, 480 and 1000 Hz were respectively 3.4, 1.8, 1.2 and 0.8 ms for flight time, and 1.4%, 0.7%, 0.5% and 0.3% for jump height. Assessed relative to either differences in jump height between elite football players (standard deviation of ~12%) or the smallest expected test-retest variability (typical error of ~3%), the technical error was substantial at 120 Hz but negligible at 240 Hz or higher. In conclusion, use of frame rates above 240 Hz to estimate jump height with MyJump will not improve accuracy substantially.

## INTRODUCTION

With the advent of smartphones, digital video cameras with high resolution and high frame rate capabilities have increasingly become available to a consumer level. Consequently, the use of mobile applications for time-based measurements with video analysis for sport sciences has risen. Through video observation, manual digitization is required from the user once the execution has been recorded to localize specific events. Examples of such events are initial and end frames in order to calculate barbell velocity on bench-press exercise [1], different markers that athletes cross while running to measure sprint performance outcomes [2], contact and take-off instants for each foot in treadmill to estimate running mechanics [3] or take-off and landing instants to compute vertical jump height from flight time [4]. All these applications take advantage of the high speed capability of current smartphones and tablets since they are cost effective and easy to use.

For the case of vertical jump height measurement, high-speed video recordings allows the user to manually select the frames closest to both take-off and landing events by visual observation. Then, the app counts the number of video frames between such events and computes flight time using frame rate duration and a basic kinematic equation [5]. One of the most popular smartphone applications, *MyJump*, has been validated with video frame rates of current smartphones, 120 Hz [6] and 240 Hz [4] and also tablets working at 60 Hz [7]. These video frames rates are a fraction of the typical sampling frequencies of 1000 Hz in laboratory-based instruments, such as force plates and jump mats [8]. Just recently, some smartphone models have been launched with very high frame rate video recording capabilities, equalizing the sampling capacity of sport sciences instrumentation. Due to the technological developments, it is expected that consumer end segment smartphone will soon include very high video sampling frame rates, similar to laboratory-based instruments. As of 2022, Sony and Samsung offer image sensors of 1280 × 720 pixels image resolution at native framerate of 960 Hz.

Authors of the first study with a smartphone capturing at 120 Hz suggested that future technical improvements might result in improved accuracy [6]. However, there are a number of drawbacks in working with ultra-high speed video frame rates. From a technical perspective, the demand for electronic devices to record, process and storage high-speed video recordings is huge [9, 10]. Also, since the main source of data is human observation, it is unclear that an increase in video frame rate would result in an effective rise of accuracy in outcomes due to hesitation in selecting the right key frame, providing the time difference between frames is low enough to look alike. In a review of the use of *MyJump*, smartphones operating at 240 Hz did not appear to offer substantial improvements in observation accuracy in jump performance [11]. The aim of this study was therefore to determine if ultra-high video speed would enhance observation accuracy. Our approach was to partition and estimate the contributions of technical error and biological variability to error of measurement in jump height at various frame rates from 120 Hz to 1000 Hz. We also used this approach to analyse and review the accuracy of *MyJump* in previous studies.

## MATERIALS AND METHODS

### Subjects

Ten healthy sport sciences students (four females, six males) volunteered to participate in the present study (age 23.1 ± 2.5 y; height 172.0 ± 8.0 cm; Body mass 68.3 ± 11.3 kg). Subjects were a sample of recreational active students with a broad range of abilities and training. None of them showed any lower-limb injury nor they were medicated. Subjects were instructed not to drink alcohol or caffeinated beverages for 24 h before testing. All jumps were performed by each subject at the same time of the day to eliminate effects of circadian rhythm. This study was approved by the Human Research Ethics Committee of the University of Alicante (IRB No. UA-2019-02-25). Each participant signed a written informed consent before participation.

### Instrumentation

The study was conducted in a sports biomechanics laboratory. A commercial high speed camera Sony DSC-RX100 IV (Sony Co., Ltd., Tokyo, Japan) was used to record both feet in frontal plane at approximately 1.5 m in order to observe take-off and landing instants, following the guidelines of *MyJump* [6]. The camera was placed on a tripod at a height of 30 cm for all recordings and the focal distance was set to adjust an optimal view of the participant’s feet. All trials were captured in high definition (1920 × 1080 pixels, progressive video) and maximum frame rate (1000 Hz), with a shutter speed of 1/32000 s. To compensate the loss of light entering the sensor when working at such high shutter speeds, two Dexel LFS-4/55 lights (Dexel, S.R.L., Buenos Aires, Argentina) where set symmetrically around the subject in the frontal plane.

**FIG. 1:**
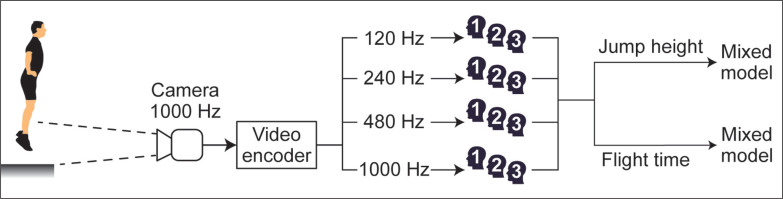
Graphical representation of the procedure. Countermovement jumps were video recorded at 1000 Hz, resampled to 120, 240, and 480 Hz, coded by three observers, and analyzed for technical errors in flight time and jump height with mixed models.

As shown in Figure 1, video recordings at 1000 Hz were transcoded to generate versions of the same footage at 120, 240 and 480 Hz by means of frame decimation with the ffmpeg command line tool (www.ffmpeg.org). Then, for all observers, videos were analyzed with *MyJump* 2 (v.6.1.5) on the same MacBook Air laptop (Apple Inc., California, USA) with 13.3” screen and 1440 × 900 pixels resolution.

### Procedures

This was an observational study consisting of repeated measurements on participants during a single testing session. After a standardized warm-up period of 5 min on a cycle ergometer set up at 80 W power load [12], the subjects performed several familiarization jumps followed by five repetitions of countermovement jumps (CMJ) with a rest period of 1 min between jumps. Subjects flexed knees to an angle of 90 degrees, which was controlled by real-time video analysis in the sagittal plane through digitizing software, and jumped to maximum effort in a continuous movement with hands on hips. If subjects failed to follow the above guidelines, they were instructed to repeat any jump performed incorrectly.

Once all executions were recorded and processed to obtain the four frame rate videos, jump height was assessed by three independent observers with past experience of *MyJump*. Before assessment, observers performed a training phase to agree on criteria when selecting video key frames: the first frame displaying both feet off the ground as take-off instant and at least one foot touching the ground as landing instant [13]. The difference between these time events is flight time. Jump height can easily be computed following the equation described in the literature [5], *h* = *t*^2^*g*/8, where *h* is jump height (m), *t* is flight time (s) and *g* is the gravity acceleration (9.81 m/s^2^). This equation is used natively in *MyJump* to display jump height on screen [6]. In all cases, observers visualized video recordings independently to each other to avoid cross influences. The videos were not analyzed with any consistent ordering of the frame rates or participants.

### Statistical Analyses

Jump height and flight time were analyzed separately with mixed linear models, realized with Proc Mixed in the Statistical Analysis System (version 9.4, SAS Institute, Cary NC, USA). The measurements for the four frame rates by the three observers were effectively simultaneous, allowing measurement error for each jump to be partitioned into subject variability and technical error. The fixed effects were trial (five levels), observer (three levels), frame rate (four levels), and the interaction of trial and frame rate (to estimate the means for each jump and the mean paired differences between frame rates and between observers). The random effects, estimated as variances and expressed as SDs, were the identity of the participant (to estimate true mean differences between subjects), the interaction of trial with identity of the participant (to estimate the variability arising from the participant for each jump), and a different residual random error for each of the four frame rates (to estimate the technical error arising from each frame rate for each jump). Jump height was log-transformed for the analysis, and effects and errors were back-transformed to percent units. Flight time was expressed in milliseconds and was analyzed without transformation.

Previous studies of the accuracy of jump height with *MyJump* did not use mixed modeling to extract components of variance, but comparisons of jump height measured with flight time via *MyJump* and either a contact platform or a force plate should yield an estimate of the technical error with *MyJump*, on the basis of the reasonable assumption that the technical error with a contact platform or a force plate is negligible in comparison with that of *MyJump*. For comparison with our estimates of technical error, we derived the technical error from published studies by three methods: the SD of the difference scores (sometimes estimated from the limits of agreement in a Bland-Altman plot) [4, 6, 14–16]; the standard error of the estimate, derived by combining the between-subject SD with a Pearson correlation [4, 6, 14, 15] or an intraclass correlation [16] between the flight times; and the standard error of measurement, derived by combining the between-subject SD with an intraclass correlation co-efficient (ICC) for two raters evaluating the same videos [15] or for one rater re-evaluating the same videos [4]. The correlations were often shown as 0.99, which results in considerable uncertainty in the estimate of the technical error, depending on whether the actual value was 0.985, 0.994, or even 0.999 truncated to 0.99, so we used estimates derived from such correlations only as a check on those derived from the difference scores.

Magnitudes of differences between means and of the technical errors were evaluated using standardization, by dividing the difference and errors by an appropriate between-subject SD. Sampling uncertainty in effects and SD is shown as 90% compatibility limits (90%CL), derived by assuming a normal sampling distribution (*t* for effects, *z* for random-effect SDs squared, and chi-squared for residual variances; 90%CL shown in ± form for SD and ×/÷ form for residuals are approximate). Decisions about magnitudes accounting for the uncertainty were based on one-sided interval hypothesis tests, where an hypothesis of a given magnitude (substantial, non-substantial) was rejected if the 90% compatibility interval fell outside that magnitude [17]. The *p* value for a test was the area of the sampling distribution of the effect (*t* for means, *z* for variances) falling in the hypothesized magnitude, with the distribution centred on the observed effect. Hypotheses of inferiority (substantial negative) and superiority (substantial positive) were rejected if their respective *p* values (*p*_–_ and *p*_+_) were < 0.05; rejection of both hypotheses represents a decisively trivial effect in equivalence testing. Hypotheses of non-inferiority (non-substantial-negative) or non-superiority (non-substantial-positive) were rejected if their respective p values (*p*_N–_ = 1 – *p*_–_, *p*_N+_ = 1 – *p*_+_) were < 0.05, representing decisively substantial effects in minimum-effects testing. The area of the sampling distribution falling in substantial or trivial magnitudes was also interpreted as the posterior probability of a substantial true magnitude of the effect in a reference-Bayesian analysis with a minimally informative prior [18] using the following scale: > 0.25, possibly; > 0.75, likely; > 0.95, very likely; > 0.995, most likely [18]. Probabilities were not interpreted for unclear effects: those with inadequate precision at the 90% level, defined by failure to reject both substantial hypotheses (*p*_–_ > 0.05 and *p*_+_ > 0.05).

The between-subject SD chosen for standardizing was not that of the subjects in our study, since they do not represent a sample of a population of competitive athletes. Instead, we obtained SD from recent studies on CMJ height of elite male [19–22] and female [23–26] football players. The SD were expressed as coefficients of variation (CV), converted to factors, log-transformed, averaged (via equally weighted variances) and back-transformed.

The technical error in jump height at a given frame rate was also evaluated for magnitude by consideration of how it adds to the biological variability in jump height to give the typical error of measurement. Again, in respect of biological variability, the subjects in the present study are not representative of the kind of competitive athletes who are routinely monitored for jump height. The subjects in one study were competitive athletes [14], and this study also provided the typical error. The typical errors of jump height of athletes in an earlier review [11] were also considered.

## RESULTS

The between-athlete SD in studies of elite football players were 14% and 10% for females and males, and the mean was 12%. Magnitude thresholds for small and moderate mean effects, given by 0.2 and 0.6 times the mean CV (via logs), were 2.3% and 7.1% respectively. The thresholds for standard deviations were half those for means [27], 1.2% and 3.5%.

Jump height across all trials, observers and frame rates was 31.7 ± 5.6 cm (mean ± SD). Differences between mean jump heights were most likely trivial for every pairwise comparison of frame rate, the biggest difference being 0.1% (90%CL ± 0.1%; rejection of both substantial hypotheses, *p* < 0.005). The three pairwise differences between means for observers at each frame rate were also most likely trivial (rejection of both substantial hypotheses, *p* < 0.005), the biggest differences for frame rates of 120, 240, 480 and 1000 Hz being respectively 1.7% (± 0.5%), 1.4% (± 0.2%), 1.1% (± 0.2%), and 0.8% (± 0.1%). Differences between means for trials were also all trivial, but all were unclear (rejection of no substantial hypotheses, *p* > 0.05), the biggest difference being 2.1% (± 4.6%), for the last minus the first trial.

The random effects in the mixed model provided estimates for pure differences in jump height between subjects of 19% (90%CL ± 10%) and pure within-subject variability between jumps of 6.4% (± 1.3%). These SD relate to the ability and reproducibility of jump performance of the subjects, but they are not relevant to the evaluation of the technical error at each frame rate provided by the residuals.

Plots of residuals vs predicteds from the mixed models for jump height and flight time showed no evidence of non-uniformity. The mixed model did not permit separate estimation of technical error for each observer, but the SD of residuals for each observer showed differences from each other that would be expected, given their sampling uncertainty, hence estimation of a single SD for technical error for the three observers was justified. Technical errors from the analysis of flight time and jump height at the four frame rates are shown in Table 1. Uncertainties in these errors evaluated relative to magnitude thresholds provided by the between-subject SD for elite football players showed that the error was small at 120 Hz and negligible at higher frame rates.

**TABLE 1:** Technical error arising from the four video frame rates.

Frame rate (Hz)	Technical error			
	(ms)	(%)	90%CL	Magnitudea
120	3.4	1.4	×/÷1.14	small*
240	1.8	0.7	×/÷1.11	trivial^00^
480	1.2	0.5	×/÷1.11	trivial^00^
1000	0.8	0.3	×/÷1.10	trivial^00^

90%CL, factor 90% confidence limits.

a Magnitude of the observed error in relation to threshold for small of 1.2% for jump height (0.1 of the SD of elite footballers). Reference-Bayesian likelihoods of true substantial magnitude:

* very likely (rejection of the non-superiority hypothesis (*p* = 0.02). Reference-Bayesian likelihoods of true trivial magnitude:

00 most likely (rejection of superiority and inferiority hypotheses (*p* < 0.005).

Table 2 summarizes data from published studies of technical error in jump height measured with *MyJump* derived from difference scores and correlation coefficients. Technical error in the only study conducted with a frame rate of 120 Hz [6] was ~4 ms (~1.5%). Frame rate in all the other studies was 240 Hz; the lowest error was 2.1 ms (0.9%), while the other errors ranged from 4.1 to 15 ms (1.5 to 7.5%). The errors were generally larger when estimated via correlation coefficients than via difference scores.

**TABLE 2:** Estimates of technical error in various units by comparison of flight time measured with *MyJump* and a jump mat or a force plate, or with repeated use of *MyJump* on the same jumps. Estimates were obtained via difference scores and via correlations. Studies are sorted in approximate order of increasing technical error. Frame rate was 120 Hz in [6] and 240 Hz in all other studies. All subjects performed countermovement jumps; subjects in two studies [4, 14] also performed drop jumps. The uncertainty in factor 90% compatibility limits (90%CL) applies to both measures and all units of technical error.

Study	Subjects	Jumps	Data	Mean ± SD (cm)	TE via difference scores				*r* or ICC	TE via *r* or ICC			
					(cm)	(%)	(ms)	90%CL		(cm)	(%)	(ms)
Carlos-Vivas et al., 2018 [16]	29 M & 11 F	5	FP	28.7 ± 7.2	0.3	0.9	2.1	×/÷1.10	1.00^a^	-	-	-

Balsalobre-Femández et al., 2015 [6]	20 M	5	Day 1, JM	35.2 ± 5.4	0.5	1.4	3.8	×/÷1.14	0.995	0.5	1.5	4.1

Gallardo-Fuentes et al., 2016 [14]	14 M athletes	5	Day 1, JM	40.0 ± 6.9	0.6	1.5	4.3	×/÷1.18	0.99	1.0	2.4	6.9
			Day 2, JM	39.2 ± 6.7	0.7	1.8	5.0	×/÷1.18	0.99	1.0	2.4	6.8

	14 M athletes (drop jump)	5	Day 1, JM	33.2 ± 5.4	0.8	2.4	6.3	×/÷1.18	0.99	0.8	2.3	6.0
			Day 2, JM	33.5 ± 6.2	0.7	2.1	5.5	×/÷1.18	0.99	0.9	2.6	6.8

Stanton et al., 2017 [4]	19 F & 10 M (drop jump)	2	Day 1, FP	19.4 ± 8.4	0.6	3.3	6.5	×/÷1.25	0.999	0.6	3.3	6.5
			Day 1 & 8^b^	19.2 ± 8.4	0.4	2.0	4.0	×/÷1.25	0.99	1.2	6.2	12

Gallardo-Fuentes et al., 2016 [14]	7 F athletes	5	Day 1, JM	28.4 ± 5.7	0.9	3.2	7.6	×/÷1.27	0.98	1.1	4.0	9.6
			Day 2, JM	29.2 ± 5.8	1.4	4.8	12	×/÷1.27	0.97	1.4	4.8	12

	7 F athletes (drop jump)	5	Day 1, JM	27.9 ± 2.9^b^	0.7	2.5	6.0	×/÷1.27	0.98	-	-	-
			Day 2, JM	27.7 ± 4.7	0.7	2.5	6.0	×/÷1.27	0.97	1.1	4.1	9.8

Stanton et al., 2017 [4]	19 F & 10 M	2	Day 1, FP	20.6 ± 8.5	1.5	7.5	15	×/÷1.25	0.998	0.8	4.1	8.4
			Day 1 & 8^c^	20.3 ± 8.4	0.5	2.5	5.1	×/÷1.25	0.99	1.2	5.9	12

Driller et al., 2017 [15]	30 M & 31 F	2	Obs 1 & 2^d^	26.8 ± 7.5	-	-	-	×/÷1.16	0.97	1.3	4.9	11

SD, standard deviation; TE, technical error; 90%CL, factor 90% compatibility limits; r, Pearson correlation coefficient; ICC, intraclass correlation coefficient; M, Male; F, Female; FP, Force plate; JM, Jump mat; Obs, observer.

a Value provided by authors does not permit estimation of TE.

b SD is too low, presumably a typographical error; TE via r therefore not estimated.

c Jumps re-analyzed by same observer one week later.

d Jumps analyzed by two observers; data not provided for difference scores.

## DISCUSSION

The aim of this study was to evaluate errors in jump height estimated from videos at different frame rates with *MyJump*. Assessed against smallest important differences in jump height defined by standardization with elite footballers, differences in the mean jump height between video frame rates were trivial, indicating no systematic error in using any of the video frame rates. There were also trivial differences in mean height between the three observers for all the frame rates. The technical error was trivial at frame rates above 120 Hz.

A smallest important difference of 2.3% in jump height was calculated from published studies of elite male and female football players performing CMJ tests with a mean between-subject standard deviation of 12%. Although the SD for the differences between subjects in our study was higher (19%), the selection of the smallest important for elite football players is appropriate, since the aim was to test the ability of the instrument to monitor changes in jump performance for such athletes.

Although ours is the first study to estimate technical error in jump height from videos with a range of frame rates, we can compare our findings with those from studies using only one frame rate. In the only previous study that was performed with a frame rate of 120 Hz [6], the estimates of technical error in time or percent units were similar to ours (~4 ms, ~1.5%). Frame rate in the other studies [4, 14–16] was 240 Hz, but the errors were similar to ours in only one study [16] (~2 ms, ~1%) and were otherwise at least 50% and mostly several times larger. The largest error (15 ms, 7.5%) in the study of Stanton et al. [4] seems unrealistic and may have been due to a typographical or computational error. The tendency for higher values via correlation coefficients probably reflects authors providing insufficient decimal places for correlations of ~0.99 and/or truncating the correlations (e.g., reporting 0.997 as 0.99). The uncertainty in the estimates of technical error expressed as factor 90% compatibility limits ranges from ×/÷1.10 to ×/÷1.27, which is insufficient for sampling variation to explain the wide range in technical error between studies. The only plausible explanation for the wide range is that detection of the frames representing take-off and landing is problematic on some smartphones or in some settings, depending on the disposition of the phone, the subject, and lighting [9].

Assessed relative to thresholds for errors derived from the between-subject SD for elite football players, the technical error of jump height using *MyJump* with a frame rate of 240 Hz is substantial in most settings. However, the technical error can also be assessed by consideration of the extent to which it contributes to the test-retest typical error. The lowest typical errors in the *MyJump* studies were 3.1% via flight time with a force platform, 3.3% via flight time with *MyJump*, and 3.6% via take-off velocity with a force platform, for 29 male and 11 female recreationally active students [16]. *MyJump* provided similar values of 3.4% and 3.6% for two different raters of 20 active men [6]. Larger values of 3.9% and 4.5% were obtained for 14 male and seven female athletes respectively with *MyJump*, and 3.9% and 6.3% respectively via flight time with a contact platform [14]; this study also produced estimates for 1-week test-retest errors of 4.6% and 7.5% for males and females with *MyJump*, and 4.6% and 7.8% with the contact platform. Values for the typical error of measurement of various jumps with various times between jumps ranged from ~3.5% to ~5.0% in an early review [11]. We conclude that the smallest error of measurement likely to be encountered when testing athletes is ~3.0%. The technical error of 1.4% we and one other group [6] observed with a frame rate of 120 Hz would increase this typical error to √(3.0^2^ + 1.4^2^) = 3.3%. While this increase may seem negligible, it would represent the need to increase sample size or number of repeated measurements by a factor of (3.3/3.0)^2^ = 1.21 or 21% to get the same precision of the estimate of a mean change score. We therefore recommend use of 240 Hz, since the technical error of 0.7% will add to a typical error of 3.0% to give a negligible increase to 3.1%.

Our study has shown that, under presumably near-optimal conditions of video assessment, the contribution of technical error arising from the frame rate itself at 240 Hz and higher frame rates is negligible. The larger technical errors observed in some settings would therefore not be reduced by increasing the frame rate. Instead, researchers or practitioners should increase the number of repeated jumps that each athlete performs on each testing occasion, then average the values, with the aim of reducing the error below the smallest important derived from the between-athlete SD. For example, with an SD of 12% for elite football players, the smallest important error is 1.2% (0.1xSD). If the typical error between repeated jumps when testing these athletes with *MyJump* (or indeed with any other device) is 4.0%, then the number of jumps *n* needed to give an error less than 1.2% is given by 4.0/√*n* < 1.2, i.e., *n* > 11. It is important to understand that increasing the number of jumps in a given testing session will not reduce any increase in test-retest error arising from real changes in individuals *between* sessions days or weeks apart, but it will improve the precision of the estimates of such changes in each individual.

The main limitation of this study is the use of only three observers. Although there were trivial differences in mean height between the three observers for all the frame rates, a study with a larger number of observers using *MyJump* is needed to determine whether a substantial proportion of observers have systematic bias. The sample size of 10 participants was not in itself a limitation, because the large number of jumps performed and analyzed resulted in useful conclusions about the technical error at each frame rate. However, it is possible that differences in jumping styles between participants could result in perceptible differences in deciding which frames show the beginning and end of contact with the floor. Further study of technical error with athletes, rather than sport science students, would resolve this issue.

## CONCLUSIONS

Coaches and trainers can use a video frame rate of 240 Hz with *MyJump* to monitor athletes’ countermovement jump height. The use of high speed video recordings above 240 Hz for manual digitizing is unnecessary, because the technical error due purely to frame rate is negligible in comparison with the day-to-day biological variability in jump height and technical errors of up to 5% arising from *MyJump*. The contributions of technical error and biological variability to error of measurement of jump height can be reduced by averaging performance of multiple jumps.
